# Some results of Heron mean and Young’s inequalities

**DOI:** 10.1186/s13660-018-1765-0

**Published:** 2018-07-13

**Authors:** Changsen Yang, Yonghui Ren

**Affiliations:** College of Mathematics and Information Science, Henan Normal University, Xinxiang, China

**Keywords:** 15A15, 15A42, 15A60, 47A30, Heron mean, Young’s inequality, Operator inequality, Unitarily invariant norm

## Abstract

In this paper, we will show some improvements of Heron mean and the refinements of Young’s inequalities for operators and matrices with a different method based on others’ results.

## Introduction

For two positive numbers *a*, *b* and $v\in[0,1]$, the quantity

1.1 $$ F_{v}(a,b)=(1-v)\sqrt{ab}+v\frac{a+b}{2} $$

is called Heron mean. And the inequality

1.2 $$ a^{v}b^{1-v} \leq va+(1-v)b,\quad a,b > 0\text{ and }0 \leq v \leq1 $$

is called Young’s inequality. Even though these inequalities look very simple, they have attracted many researchers in this field, where adding a positive term to refine the inequalities is possible.

Heron mean is the interpolation between arithmetic and geometric means for $a,b\geq0$ and $v\in[0,1]$. We can see papers [1, 2], and [3] for some new results about Heron mean and arithmetic–geometric mean.

The first refinements of Young’s inequality is the squared version proved in [4]

1.3 $$ \bigl(a^{v}b^{1-v}\bigr)^{2}+\min\{ v,1-v \} ^{2} (a-b)^{2} \leq \bigl(va+(1-v)b\bigr)^{2}. $$

Later, the authors in [5] obtained the other interesting refinement

1.4 $$ a^{v}b^{1-v}+\min\{ v,1-v \} (\sqrt{a}- \sqrt {b})^{2} \leq va+(1-b). $$

A common fact about refinements (1.2) and (1.3) is having one refining term.

In the recent paper [6], some reverses and refinements of Young’s inequality were presented. It was proved that

1.5 $$ \textstyle\begin{cases} a^{v}b^{1-v}+v(\sqrt{a}-\sqrt{b})^{2}+r_{1}(\sqrt[4]{ab}-\sqrt {b})^{2}\leq va+(1-v)b,& 0\leq v\leq\frac{1}{2},\\ a^{v}b^{1-v}+(1-v)(\sqrt{a}-\sqrt{b})^{2}+r_{1}(\sqrt[4]{ab}-\sqrt {a})^{2}\leq va+(1-v)b,&\frac{1}{2}\leq v\leq1, \end{cases} $$

where $r_{1}=\min\{ 2r,1-2r\}$ for $r=\min\{ v,1-v\}$. In the same paper, the following reversed versions were proved:

1.6 $$ \textstyle\begin{cases}va+(1-v)b+r_{1}(\sqrt[4]{ab}-\sqrt{a})^{2}\leq a^{v}b^{1-v}+(1-v)(\sqrt{a}-\sqrt{b})^{2},& 0\leq v\leq\frac {1}{2},\\ va+(1-v)b+r_{1}(\sqrt[4]{ab}-\sqrt{b})^{2}\leq a^{v}b^{1-v}+v(\sqrt {a}-\sqrt{b})^{2},&\frac{1}{2}\leq v\leq1, \end{cases} $$

where $r_{1}=\min\{2r,1-2r\}$ for $r=\min\{v,1-v\}$.

In this paper, our main results are to give refinements of Heron mean for scalars and matrices in Sect. 2; and in Sect. 3, in a different way, to get an operator version of (1.5), which is the refinement of (1.4). Besides, in the same section, the refinements of Young’s inequalities for the Hilbert–Schmidt norm will be presented using the same technology as in Sect. 2.

For our convenience, we firstly give some denotations.

Throughout the paper, *H* is a Hilbert space and $B(H)$ denotes the set of all bounded linear operators on a complex Hilbert space *H*. An operator $A\in B^{+}(H)$ is positive invertible if $(Ax,x)>0$ for every vector $x\in H\setminus\{0\}$. $M_{n}$ denotes the space of all $n\times n$ complex matrices. The Hilbert–Schmidt norm of $A=[a_{ij}]\in M_{n}$ is defined by

$$\Vert A \Vert _{2}=\sqrt{{\sum _{i,j=1}^{n}} \vert a_{ij} \vert ^{2}}. $$

It is well known that the Hilbert–Schmidt norm is unitarily invariant in the sense that $|\!|\!|UAV |\!|\!|= |\!|\!|A|\!|\!|$ for all unitary matrices $U, V\in M_{n}$. What is more, we define

$$\begin{gathered} A\nabla_{v} B= (1-v)A+vB,\quad v\in[0,1], \\ A\sharp_{v} B=A^{\frac{1}{2}}\bigl(A^{-\frac{1}{2}}BA^{-\frac {1}{2}} \bigr)^{v}A^{\frac{1}{2}},\quad v\in R\end{gathered} $$

denoted by $A \nabla B$ and $A\sharp B$, respectively, when $v=\frac{1}{2}$.

## Main results

### Refinements of Heron mean

Heron mean is defined by

2.1 $$ F_{v}(a,b)=(1-v)\sqrt{ab}+v\frac{a+b}{2}. $$

It is easy to see that $F_{v}(a,b)$ is an increasing function in *v* on $[0,1]$ and

2.2 $$ \sqrt{ab}=F_{0}(a,b)\leq F_{v}(a,b)\leq F_{1}(a,b)= \frac{a+b}{2}. $$

Our purpose of this section is to give refinements of Heron mean for a scalar and some other auxiliary results.

#### Theorem 2.1

*For* $a,b\geq0$, *and* $v\in[0,1]$, *we have*

2.3 $$\begin{aligned}& \frac{1}{2}v(1-v) (\sqrt{a}-\sqrt{b})^{2}+\sqrt {ab}\leq F_{v}(a,b), \end{aligned}$$

2.4 $$\begin{aligned}& F_{v}(a,b)\leq\frac{a+b}{2}-\frac{1}{2}v(1-v) (\sqrt {a}- \sqrt{b})^{2}, \end{aligned}$$

*where* $F_{v}(a,b)=(1-v)\sqrt{ab}+v\frac{a+b}{2}$.

#### Proof

Firstly,

$$\begin{gathered} F_{v}(a,b)-\frac{1}{2}v(1-v) ( \sqrt{a}-\sqrt{b})^{2} \\ \quad=\frac{1}{2}(va+vb+2\sqrt{ab}-2v\sqrt{ab})-\frac {1}{2} \bigl(va+vb-2v\sqrt{ab}-v^{2}a-v^{2}b+2v^{2} \sqrt{ab}\bigr) \\ \quad=v^{2}\biggl(\frac{a+b}{2}-\sqrt{ab}\biggr)+\sqrt{ab} \\ \quad\geq\sqrt{ab}. \end{gathered} $$

Next, we also have

$$\begin{gathered} \frac{a+b}{2}-\frac{1}{2}v(1-v) (\sqrt{a}- \sqrt{b})^{2}-F_{v}(a,b) \\ \quad=\frac{1}{2}\bigl(a+b-va-vb+2v\sqrt{ab}+v^{2}a+v^{2}b-2v^{2} \sqrt {ab}-va-vb-2\sqrt{ab}+2v\sqrt{ab}\bigr) \\ \quad=\frac{1}{2}\bigl[(1-2v)a+(1-2v)b-2(1-2v)\sqrt{ab}+v^{2}(a+b-2 \sqrt {ab})\bigr] \\ \quad=\frac{1}{2}(1-v)^{2}(\sqrt{a}-\sqrt{b})^{2} \\ \quad\geq0. \end{gathered} $$

 □

It is clear that $\frac{1}{2}v(1-v)(\sqrt{a}-\sqrt{b})^{2}\geq0$, so (2.3) and (2.4) are refinements of (2.2).

With Theorem 2.1 in hand, we will give refinements of Heron mean for operators by the monotonicity property of operator functions.

#### Lemma 2.2

*Let* $X\in B(H)$ *be self*-*adjoint*, *and let* *f* *and* *g* *be continuous real functions such that* $f(t)\geq g(t)$ *for all* $t\in \operatorname{Sp}(X)$ (*the spectrum of* *X*). *Then* $f(X)\geq g(X)$.

For more details about this property, the reader is referred to [7].

#### Theorem 2.3

*Let* $A,B\in B^{+}(H)$ *be positive invertible operators*, *I* *be the identity operator*, *and* $v\in[0,1]$, *then we have*

2.5 $$ v(1-v) (A\bigtriangledown B-A\sharp B)+A\sharp B\leq F_{v}(A,B) $$

*and*

2.6 $$ F_{v}(A,B)\leq A\bigtriangledown B-v(1-v) (A\bigtriangledown B-A\sharp B), $$

*where* $F_{v}(A,B)=vA\bigtriangledown B+(1-v)A\sharp B$.

#### Proof

Let $b=1$ in (2.3) and expand the summand to get

2.7 $$ \frac{1}{2}v(1-v) (a+1-2\sqrt{a})+\sqrt{a}\leq\frac {1}{2}v(a+1)+(1-v) \sqrt{a}. $$

Note that the operator $X=A^{-\frac{1}{2}}BA^{-\frac{1}{2}}$ has a positive spectrum, and by Lemma 2.2 and (2.7) we have

2.8 $$ \frac{1}{2}v(1-v) \bigl(X+I-2X^{\frac{1}{2}}\bigr)+X^{\frac {1}{2}}\leq \frac{1}{2}v(X+I)+(1-v)X^{\frac{1}{2}}. $$

Finally, multiplying inequality (2.8) by $A^{\frac{1}{2}}$ on the left- and right-hand sides, we can get

$$\frac{1}{2}v(1-v) (A+B-2A\sharp B)+A\sharp B\leq\frac {1}{2}v(A+B)+(1-v)A \sharp B, $$

which is equivalent to (2.5).

Using the same technique in (2.4), we can get (2.6). So we completed the proof. □

Next, we will present the refinements of Heron mean for the Hilbert–Schmidt norm.

#### Theorem 2.4

*Suppose* $A,B,X\in M_{n}$ *such that* *A*, *B* *are two positive definite matrices for* $0\leq v\leq1$, *then we have*

2.9 $$ \begin{aligned}[b] &\frac{1}{2}v(1-v)|\!|\!|AX-XB |\!|\!|^{2}_{2}+\big|\!\big|\!\big|A^{\frac {1}{2}}XB^{\frac{1}{2}} \big|\!\big|\!\big|^{2}_{2} \\ &\quad\leq\frac{1}{2}v|\!|\!|AX+XB|\!|\!|^{2}_{2}+(1-2v) \big|\!\big|\!\big|A^{\frac{1}{2}}XB^{\frac{1}{2}}\big|\!\big|\!\big|^{2}_{2} \\ &\quad\leq\frac{1}{2}|\!|\!|AX+XB|\!|\!|^{2}_{2}- \frac {1}{2}v(1-v)|\!|\!|AX-XB|\!|\!|^{2}_{2}-\big|\!\big|\!\big|A^{\frac {1}{2}}XB^{\frac{1}{2}}\big|\!\big|\!\big|^{2}_{2}. \end{aligned} $$

#### Proof

Replace *a*, *b* by $a^{2}$, $b^{2}$ in (2.3) and (2.4), then we have

2.10 $$ \begin{aligned}[b] &\frac{1}{2}v(1-v) (a-b)^{2}+ \bigl(a^{\frac{1}{2}}b^{\frac{1}{2}}\bigr)^{2} \\ &\quad\leq\frac{1}{2}v(a+b)^{2}+(1-2v) \bigl(a^{\frac{1}{2}}b^{\frac {1}{2}} \bigr)^{2} \\ &\quad\leq\frac{1}{2}(a+b)^{2}-\frac{1}{2}v(1-v) (a-b)^{2}-\bigl(a^{\frac {1}{2}}b^{\frac{1}{2}}\bigr)^{2}. \end{aligned} $$

Since *A* and *B* are positive definite, it follows by the spectral theorem that there exist unitary matrices $U,V\in M_{n}$ such that

$$A=U\Lambda_{1}U^{\ast},\qquad B=V\Lambda_{2}V^{\ast}, $$

where $\Lambda_{1}=\operatorname{diag}(\lambda_{1},\lambda_{2},\ldots,\lambda _{n})$, $\Lambda_{2}=\operatorname{diag}(\nu_{1},\nu_{2},\ldots,\nu_{n})$, $\lambda _{i},\nu_{i}>0$, $i=1,2,\ldots,n$.

Let $Y=U^{\ast}XV=[y_{il}]$, then

$$\begin{gathered} AX-XB=U\bigl[(\lambda_{i}-\nu_{l})y_{il} \bigr]V^{\ast}; \\ A^{\frac{1}{2}}XB^{\frac{1}{2}}=U\bigl[\bigl(\lambda_{i}^{\frac {1}{2}} \nu_{l}^{\frac{1}{2}}\bigr)y_{il}\bigr]V^{\ast};\end{gathered} $$

and

$$AX+XB=U\bigl[(\lambda_{i}+\nu_{l})y_{il} \bigr]V^{\ast}. $$

Now, by (2.10) and the unitary invariance of the Hilbert–Schmidt norm, we have

$$\begin{gathered} \frac{1}{2}v(1-v)|\!|\!|AX-XB |\!|\!|^{2}_{2}+\big|\!\big|\!\big|A^{\frac {1}{2}}XB^{\frac{1}{2}} \big|\!\big|\!\big|^{2}_{2} \\ \quad={\sum_{i,l=1}^{n}}\biggl\{ { \frac{1}{2}v(1-v) (\lambda_{i}-\nu _{l})^{2}+ \bigl(\lambda_{i}^{\frac{1}{2}}\nu_{l}^{\frac{1}{2}} \bigr)^{2}}\biggr\} \vert y_{il} \vert ^{2} \\ \quad\leq{\sum_{i,l=1}^{n}}\biggl\{ \frac{1}{2}v(\lambda_{i}+\nu _{l})^{2}+(1-2v) \bigl(\lambda_{i}^{\frac{1}{2}}\nu_{l}^{\frac {1}{2}} \bigr)^{2}\biggr\} \vert y_{il} \vert ^{2} \\ \quad=\frac{1}{2}v|\!|\!|AX+XB|\!|\!|^{2}_{2}+(1-2v) \big|\!\big|\!\big|A^{\frac {1}{2}}XB^{\frac{1}{2}}\big|\!\big|\!\big|^{2}_{2} \\ \quad\leq{\sum_{i,l=1}^{n}}\biggl\{ \frac{1}{2}(\lambda_{i}+\nu _{l})^{2}- \frac{1}{2}v(1-v) (\lambda_{i}-\nu_{l})^{2}- \bigl(\lambda _{i}^{\frac{1}{2}}\nu_{l}^{\frac{1}{2}} \bigr)^{2}\biggr\} \vert y_{il} \vert ^{2} \\ \quad=\frac{1}{2}|\!|\!|AX+XB|\!|\!|^{2}_{2}- \frac{1}{2}v(1-v)|\!|\!|AX-XB|\!|\!|^{2}_{2}-\big|\!\big|\!\big|A^{\frac{1}{2}}XB^{\frac{1}{2}}\big|\!\big|\!\big|^{2}_{2}. \end{gathered} $$

So we finished the proof. □

### Refinements of Young’s inequalities

It is well known that

2.11 $$ a^{1-v}b^{v} \leq(1-v)a+vb,\quad a,b > 0\text{ and }v\in[0,1] $$

with equality if and only if $a=b$ is called Young’s inequality.

An operator version of (2.11) in [7] says that

2.12 $$ A\sharp_{v}B\leq A\nabla_{v} B $$

for $A,B\in B^{+}(H)$ and $v\in[0,1]$. Kittaneh and Manasrah [5] gave a different type of improvement of Young’s matrix inequalities:

2.13 $$ 2r(A\nabla B-A\sharp B)\leq A\nabla_{v} B-A\sharp _{v}B \leq2s(A\nabla B-A\sharp B) $$

for $A, B\in B^{+}(H)$, $v\in[0,1]$, $r=\min\{v, 1-v\}$, and $s=\max \{v, 1-v\}$.

Here, we give the first inequalities’ refinements of (2.13). Before that, we need a lemma.

#### Lemma 2.5

([8])

$$A\nabla_{\mu}(A\sharp_{\nu}B)=A\nabla_{\mu\nu}B-\mu(A \nabla _{\nu}B-A\sharp_{\nu}B) $$

*for* $0\leq\mu, \nu\leq1$ *and* *A*, *B* *are positive operators*.

#### Proof

$$\begin{gathered} A\nabla_{\mu}(A\sharp_{\nu}B) \\ \quad=(1-\mu)A+\mu A\sharp_{\nu}B \\ \quad=A+\mu\nu B-\mu\nu A-\mu\bigl[(1-\nu)A+\nu B-A\sharp_{\nu}B\bigr] \\ \quad=A\nabla_{\mu\nu}B-\mu(A\nabla_{\nu}B-A\sharp_{\nu}B). \end{gathered} $$

 □

#### Theorem 2.6

1. *If* $0\leq\nu\leq\frac{1}{2}$, *then*
  2.14 $$ 2r_{1}(A\nabla_{\frac{1}{4}}B-A\sharp_{\frac {1}{4}}B)+(2r-r_{1}) (A\nabla B-A\sharp B)\leq A\nabla_{v} B-A\sharp _{v}B. $$
2. *If* $\frac{1}{2}\leq\nu\leq1$, *then*
  2.15 $$ 2r_{1}(A\nabla_{\frac{3}{4}}B-A\sharp_{\frac {3}{4}}B)+(2r-r_{1}) (A\nabla B-A\sharp B)\leq A\nabla_{v} B-A\sharp _{v}B, $$
  *where* $r=\min\{\nu, 1-\nu\}$ *and* $r_{1}=\min\{2r, 1-2r\}$.

#### Proof

For $0\leq\nu\leq\frac{1}{2}$, then we have $0\leq2\nu\leq1$. Substituting *B* by $A\sharp B$ and *ν* by 2*ν* in the first inequality (2.13), we have

2.16 $$ 2\min\{2r, 1-2r\}\bigl(A\nabla(A\sharp B)-A\sharp(A\sharp B)\bigr)\leq A \nabla_{2v} (A\sharp B)-A\sharp_{2v}(A\sharp B). $$

By computing directly with Lemma 2.5, then we have

2.17 $$ 2r_{1}(A\nabla_{\frac{1}{4}}B-A\sharp_{\frac {1}{4}}B)+(2r-r_{1}) (A\nabla B-A\sharp B)\leq A\nabla_{v} B-A\sharp _{v}B. $$

Exchanging *A* for *B* and *ν* for $1-\nu$ in (2.17) for $\frac {1}{2}\leq\nu\leq1$, we get

2.18 $$ 2r_{1}(A\nabla_{\frac{3}{4}}B-A\sharp_{\frac {3}{4}}B)+(2r-r_{1}) (A\nabla B-A\sharp B)\leq A\nabla_{v} B-A\sharp _{v}B. $$

So we completed the proof. □

#### Remark 2.7

Our inequalities (2.14) and (2.15) are stronger than the first inequality (2.13), that is,

1. for $0\leq\nu\leq\frac{1}{2}$,
  2.19 $$ 2r(A\nabla B-A\sharp B)\leq2r_{1}(A\nabla_{\frac {1}{4}}B-A \sharp_{\frac{1}{4}}B)+(2r-r_{1}) (A\nabla B-A\sharp B); $$
2. for $\frac{1}{2}\leq\nu\leq1$,
  2.20 $$ 2r(A\nabla B-A\sharp B)\leq2r_{1}(A\nabla_{\frac {3}{4}}B-A \sharp_{\frac{3}{4}}B)+(2r-r_{1}) (A\nabla B-A\sharp B), $$
  where $\nu\in[0, 1]$, $r=\min\{\nu, 1-\nu\}$ and $r_{1}=\min\{2r, 1-2r\}$.

#### Proof

For $0\leq\nu\leq\frac{1}{4}$, then $r=\nu$, $r_{1}=2\nu$. So (2.19) is equivalent to

2.21 $$ 4\nu(A\nabla_{\frac{1}{4}}B-A\sharp_{\frac {1}{4}}B)\geq2\nu(A\nabla B-A\sharp B), $$

that is,

2.22 $$ \frac{3}{2}A+\frac{1}{2}B-2A^{\frac{1}{2}}\bigl(A^{-\frac {1}{2}}BA^{-\frac{1}{2}} \bigr)^{\frac{1}{4}}A^{\frac{1}{2}}\geq\frac {A+B}{2}-A^{\frac{1}{2}} \bigl(A^{-\frac{1}{2}}BA^{-\frac{1}{2}}\bigr)^{\frac {1}{2}}A^{\frac{1}{2}}. $$

So we only need to prove

2.23 $$ I+\bigl(A^{-\frac{1}{2}}BA^{-\frac{1}{2}}\bigr)^{\frac {1}{2}}\geq2 \bigl(A^{-\frac{1}{2}}BA^{-\frac{1}{2}}\bigr)^{\frac{1}{4}}, $$

which is clearly true for *A*, *B* are positive definite operators.

For $\frac{1}{4}\leq\nu\leq\frac{1}{2}$, then $r=\nu$, $r_{1}=1-2\nu$. Equation (2.19) is equivalent to

2.24 $$ 2(1-2v) (A\nabla_{\frac{1}{4}}B-A\sharp_{\frac {1}{4}})+(2v-1+2v) (A\nabla B-A \sharp B)\geq2\nu(A\nabla B-A\sharp B), $$

that is,

2.25 $$ 2(A\nabla_{\frac{1}{4}}B-A\sharp_{\frac {1}{4}}B)\geq(A\nabla B-A\sharp B). $$

By (2.21), we can prove (2.25) directly.

Similarly, we can prove (2.20).

For $\frac{3}{4}\leq\nu\leq1$, then $r=1-\nu$, $r_{1}=2-2\nu$. So (2.20) is equivalent to

2.26 $$ (4-4v) (A\nabla_{\frac{3}{4}}B-A\sharp_{\frac {3}{4}}B)\geq2(1-v) (A\nabla B-A \sharp B), $$

that is,

2.27 $$ B+A^{\frac{1}{2}}\bigl(A^{-\frac{1}{2}}BA^{-\frac {1}{2}}\bigr)^{\frac{1}{2}}A^{\frac{1}{2}} \geq2A^{\frac{1}{2}}\bigl(A^{-\frac {1}{2}}BA^{-\frac{1}{2}}\bigr)^{\frac{3}{4}}A^{\frac{1}{2}}. $$

Multiplying by $A^{-\frac{1}{2}}$ on both sides and dividing by $(A^{-\frac{1}{2}}BA^{-\frac{1}{2}})^{\frac{1}{2}}$, we get

2.28 $$ \bigl(A^{-\frac{1}{2}}BA^{-\frac{1}{2}}\bigr)^{\frac {1}{2}}+I\geq2 \bigl(A^{-\frac{1}{2}}BA^{-\frac{1}{2}}\bigr)^{\frac{1}{4}}, $$

which is clearly true for *A*, *B* are positive definite operators.

For $\frac{1}{2}\leq\nu\leq\frac{3}{4}$, then $r=1-\nu$, $r_{1}=2\nu-1$. So (2.20) is equivalent to

2.29 $$ (4v-2) (A\nabla_{\frac{3}{4}}B-A\sharp_{\frac {3}{4}}B)\geq(2\nu-1) (A\nabla B-A \sharp B), $$

that is,

2.30 $$ 2(A\nabla_{\frac{3}{4}}B-A\sharp_{\frac {3}{4}}B)\geq(A\nabla B-A\sharp B), $$

which can be got directly from (2.26). □

Here, we should remind the readers that the reverse of Theorem 2.6 is stronger than (2.13) and only holds for $0\leq\nu\leq\frac{1}{4}$ and $\frac{3}{4}\leq\nu\leq1$ in Zhao and Li [8]. We also can see Zhao and Wu [6] for a different method to get Theorem 2.6.

Next, we will present refinements of Young’s inequalities (2.14) and (2.15) for the Hilbert–Schmidt norm. Firstly, we give their scalar type inequalities. That is to say, for $0\leq\nu\leq\frac{1}{2}$, we have

2.31 $$ a^{1-v}b^{v}+v(\sqrt{a}-\sqrt{b})^{2}+r_{1} \bigl(\sqrt [4]{ab}-\sqrt{a}\bigr)^{2} \leq(1-v)a+vb, $$

where $\nu\in[0, 1]$, $r=\min\{\nu, 1-\nu\}$, and $r_{1}=\min\{2r, 1-2r\}$.

Substituting *a* by $a^{2}$ and *b* by $b^{2}$ in (2.31) respectively, we get

2.32 $$ \bigl(a^{1-v}b^{v}\bigr)^{2}+v(a-b)^{2}+r_{1}( \sqrt{ab}-a)^{2} \leq(1-v)a^{2}+vb^{2}, $$

that is,

2.33 $$ \bigl(a^{1-v}b^{v}\bigr)^{2}+v(a-b)^{2}+r_{1}( \sqrt {ab}-a)^{2}+2v(1-v)ab \leq\bigl((1-v)a+vb\bigr)^{2}. $$

Similarly, for $\frac{1}{2}\leq\nu\leq1$, we have

2.34 $$ \bigl(a^{1-v}b^{v}\bigr)^{2}+(1-v) (a-b)^{2}+r_{1}(\sqrt {ab}-b)^{2}+2v(1-v)ab \leq \bigl((1-v)a+vb\bigr)^{2}. $$

Using the same method with Theorem 2.4 in (2.33) and (2.34), we can have the following results.

#### Corollary 2.8

*Suppose* $A,B,X\in M_{n}$ *such that* *A*, *B* *are two positive definite matrices*, *for* $0\leq\nu\leq\frac{1}{2}$, *we have*

2.35 $$ \begin{aligned}[b] & \bigl\Vert A^{1-v}XB^{v} \bigr\Vert _{2}^{2}+r \Vert AX-XB \Vert _{2}^{2}+r_{1} \bigl\Vert A^{\frac {1}{2}}XB^{\frac{1}{2}}-AX \bigr\Vert _{2}^{2}+2v(1-v) \bigl\Vert A^{\frac {1}{2}}XB^{\frac{1}{2}} \bigr\Vert _{2}^{2} \\ &\quad\leq \bigl\Vert (1-v)AX-vXB \bigr\Vert _{2}^{2}; \end{aligned} $$

*for* $\frac{1}{2}\leq\nu\leq1$, *we have*

2.36 $$ \begin{aligned}[b] & \bigl\Vert A^{1-v}XB^{v} \bigr\Vert _{2}^{2}+r \Vert AX-XB \Vert _{2}^{2}+r_{1} \bigl\Vert A^{\frac {1}{2}}XB^{\frac{1}{2}}-XB \bigr\Vert _{2}^{2}+2v(1-v) \bigl\Vert A^{\frac {1}{2}}XB^{\frac{1}{2}} \bigr\Vert _{2}^{2} \\ &\quad\leq \bigl\Vert (1-v)AX-vXB \bigr\Vert _{2}^{2}. \end{aligned} $$

## Discussion

In the theory of operators, the operator means and operator inequalities are two key concepts. An effective method to study operators is to find some refinements among some operator means, and these inequalities are usually based on scalars or matrices.

## Conclusion

In order to better estimate the Heron mean, a refinement inequality about the classical interpolation between arithmetic mean and geometric mean by Heron mean is obtained, which is also applicable to establishing the inequalities for operators and matrices. Next an operator version refinement inequality about Young’s inequality is also established, which is a generalization on the results obtained previously by Kittaneh and Manasrah [5]. It is worth noting that the inequality mentioned can also give the refinement inequality about Young’s inequality, which was presented by Zhao and Wu [6].
